# Biomarker Driven Antifungal Stewardship (BioDriveAFS) in acute leukaemia—a multi-centre randomised controlled trial to assess clinical and cost effectiveness: a study protocol for a randomised controlled trial

**DOI:** 10.1186/s13063-024-08272-w

**Published:** 2024-06-28

**Authors:** Lydia Flett, Radwa Abdelatif, Sarah Akhtar Baz, Samantha Brady, Belén Corbacho, Kate Common, Abbie Cowling, Caroline Fairhurst, Ellie Fitzmaurice, Shreyans Gandhi, Andrea Hilton, William Hope, Alex Howard, Joanne Laycock, Patrick Lillie, Gemma Mitchell, Adwoa Parker, Mary Peel, Laura Sheard, Jacqueline Sneddon, Thomas Taynton, Puvan Tharmanathan, David Torgerson, Han-I Wang, David Allsup, Gavin Barlow

**Affiliations:** 1 Department of Health Sciences, University of York, York, UK; 2 Patient and Public Involvement, University of York, York, UK; 3 King’s College Hospital NHS Foundation Trust, London, UK; 4 Faculty of Health Sciences, University of Hull, Hull, UK; 5 Department of Molecular and Clinical Pharmacology, University of Liverpool, Liverpool, UK; 6 Faculty of Health and Life Sciences, University of Liverpool, Liverpool, UK; 7 Hull University Teaching Hospitals NHS Trust, Hull, UK; 8 Institute for Social Marketing and Health, University of Stirling, Stirling, UK; 9 British Society for Antimicrobial Chemotherapy, Birmingham, UK; 10 Biomedical Institute for Multimorbidity, Hull York Medical School, University of Hull, Hull, UK; 11 Department of Experimental Medicine & Biomedicine, Hull York Medical School, University of York, York, UK

**Keywords:** Acute leukaemia, Galactomannan, Beta-D-Glucan, Antifungal stewardship, Invasive fungal infection, Apergillosis

## Abstract

**Background:**

Acute leukaemias (AL) are life-threatening blood cancers that can be potentially cured with treatment involving myelosuppressive, multiagent, intensive chemotherapy (IC). However, such treatment is associated with a risk of serious infection, in particular invasive fungal infection (IFI) associated with prolonged neutropenia. Current practice guidelines recommend primary antifungal (AF) prophylaxis to be administered to high-risk patients to reduce IFI incidence. AFs are also used empirically to manage prolonged neutropenic fever. Current strategies lead to substantial overuse of AFs. Galactomannan (GM) and β-D-glucan (BG) biomarkers are also used to diagnose IFI. Combining both biomarkers may enhance the predictability of IFI compared to administering each test alone. Currently, no large-scale randomised controlled trial (RCT) has directly compared a biomarker-based diagnostic screening strategy without AF prophylaxis to AF prophylaxis (without systematic biomarker testing).

**Methods:**

BioDriveAFS is a multicentre, parallel, two-arm RCT of 404 participants from UK NHS Haematology departments. Participants will be allocated on a 1:1 basis to receive either a biomarker-based antifungal stewardship (AFS) strategy, or a prophylactic AF strategy, which includes existing standard of care (SoC).

The co-primary outcomes will be AF exposure in the 12-month post randomisation and the patient-reported EQ-5D-5L measured at 12-month post randomisation. Secondary outcomes will include total AF exposure, probable/proven IFI, survival (all-cause mortality and IFI mortality), IFI treatment outcome, AF-associated adverse effects/events/complications, resource use, episodes of neutropenic fever requiring hospital admission or outpatient management, AF resistance in fungi (non-invasive and invasive) and a Desirability of Outcome Ranking.

The trial will have an internal pilot phase during the first 9 months. A mixed methods process evaluation will be integrated in parallel to the internal pilot phase and full trial, aiming to robustly assess how the intervention is delivered. Cost-effectiveness analysis will also be performed.

**Discussion:**

The BioDriveAFS trial aims to further the knowledge of strategies that will safely optimise AF use through comparison of the clinical and cost-effectiveness of a biomarker-led diagnostic strategy versus prophylactic AF to prevent and manage IFI within acute leukaemia. The evidence generated from the study will help inform global clinical practice and approaches within antifungal stewardship.

**Trial registration:**

ISRCTN11633399. Registered 24/06/2022.

**Supplementary Information:**

The online version contains supplementary material available at 10.1186/s13063-024-08272-w.

## Introduction

### Background and rationale {6a}

Acute leukaemias (AL) are life-threatening blood cancers which include the conditions acute myeloid leukaemia (AML) and acute lymphoblastic leukaemia (ALL). AL is potentially curable but treatment involves the use of myelosuppressive, multiagent, intensive chemotherapy (IC). Related conditions such as high-risk myelodysplastic syndromes (HRMDS) and transformed myeloproliferative neoplasms (tMPN) are also sometimes treated with IC with the same approach as AL. Such therapy is associated with significant toxicities which include the risk of serious infection. Of particular concern is invasive fungal infection (IFI) associated with prolonged neutropenia. The IFI invasive aspergillosis (IA) has an incidence of approximately 4–11% in the AL population with an associated case fatality rate of up to 30% [1]. Practice guidelines recommend the use of primary antifungal (AF) prophylaxis in high-risk patients to reduce the incidence of IFI [2–4]; however, other strategies have been proposed to try and limit exposure to AF medication [5].

Mould-active triazoles are commonly prescribed for AF prophylaxis but echinocandins or amphotericin-based agents are also used dependent upon the type of AL, the chemotherapy regimen deployed, and the perceived risk of IFI [4]. Antifungal medications have significant toxicities, drug-drug interactions and are associated with a subsequent increase in antifungal resistance [6].

Current management strategies for prolonged neutropenic fever include the empiric use of an AF when patients have been febrile after 72–96 h of broad-spectrum antibacterial treatment [7]. This approach is based upon the supposition that antibiotic-resistant pyrexia, or other signs of infection, may be due to undiagnosed fungal infection. However, such an empiric strategy leads to substantial overuse of AFs, with one United Kingdom (UK)-based study finding that less than 20% of patients empirically treated with AFs have IFI [8].

Fungal infection biomarkers such as galactomannan (GM) and (1,3)-β-D-glucan (BG) are used in the diagnosis of IFI, and combined use may be more predictive of IFI than the use of either test in isolation [9]. GM and BG become positive several days before clinical symptoms or signs of infection (GM 4–9 days, BG 5–11 days) [10]. In the UK, GM and BG are the most commonly used IFI biomarkers with the majority of testing performed at reference laboratories rather than locally. A consequence of such biomarker analysis at remote sites is that any gains in early detection may be offset by test turn-around-times (TAT). TAT has been improving and the UK national fungal reference laboratory now has a median internal TAT of less than 24 h [11]. Emerging technologies, including point of care tests, may reduce TAT in the future.

A large-scale randomised controlled trial (RCT) to directly compare a biomarker-based diagnostic screening strategy without AF prophylaxis to AF prophylaxis (without systematic biomarker testing) has not been conducted. However, a study for the European Organization for the Research and Treatment of Cancer and the Mycoses Study Group (EORTC/MSG) compared a GM-based screening approach to an empirical treatment strategy; in both arms patients only received fluconazole prophylaxis, which is a non-mould acting AF that does not prevent IA. The GM-based approach reduced AF usage by more than half without any difference in fungal-free survival [12].

BioDriveAFS is a multi-centre RCT that aims to compare twice-weekly combined GM and BG biomarker screening without antifungal prophylaxis, to mould-active AF prophylaxis without biomarker screening, in patients with AL, HRMDS and tMPN treated with IC. The co-primary outcomes are the proportion of patients with 3 or more days of systemic antifungal exposure and health-related quality of life (HRQoL) at 12 months.

### Objectives {7}

#### Primary objective

To conduct a multi-centre RCT to investigate whether a biomarker-based antifungal stewardship (AFS) strategy is superior to a prophylactic mould-acting AF strategy in reducing AF therapy use in patients with AL (AML/ALL), HRMDS and tMPN treated with IC, without adverse impact on HRQoL in the 12 months from trial enrolment.

#### Secondary objectives

- To conduct a 9-month internal pilot to assess trial feasibility and to optimise processes for trial continuation.
- To conduct a mixed methods process evaluation alongside the RCT, focusing on assessment of fidelity and implementation via qualitative methods and clinical data collection. Findings will inform ongoing feedback to local research teams and potential amendments to trial processes and training as appropriate; and will subsequently inform dissemination and implementation plans within the UK National Health Service (NHS) as appropriate.
- To investigate the cost-effectiveness of a biomarker driven AFS strategy compared to prophylactic mould-acting AF within the existing local standard of care (SoC).
- To develop and strengthen a sustainable training, engagement and Patient and Public Involvement (PPI) legacy along with a network of engaged stakeholders.

### Trial design {8}

BioDriveAFS is a prospective, multi-centre, two-arm, parallel group RCT to assess the clinical and cost-effectiveness of a biomarker-based AFS strategy versus a prophylactic mould-acting AF strategy, including existing SoC, in reducing AF therapy use in adult patients (≥16 years) with AL, HRMDS and tMPN treated with IC.

A mixed methods process evaluation will be performed in parallel, which will focus on fidelity to the clinical pathway and barriers and facilitators to site trial participation and intervention implementation.

An internal pilot phase will run during the first 9 months from the start of recruitment. This period will be used to assess recruitment and retention rates, and intervention fidelity, and provide guidance on optimising the trial processes.

## Methods: participants, interventions and outcomes

### Study setting {9}

NHS haematology departments in tertiary and secondary care hospitals in the UK responsible for the delivery of IC to AL, HRMDS and tMPN patients in line with national guidance. Sites must also be able to currently have or ascertain access to GM and BG testing either internally or externally.

### Eligibility criteria {10}

#### Inclusion criteria

To be eligible for the trial, patients must meet all of the following criteria:

1. Aged ≥16 years
2. Diagnosis of new, or relapsed, AL or haematological disorder judged to need IC by the patient’s clinical care team. Eligible conditions include AML, ALL, HRMDS or tMPN
3. The patient is expected to have prolonged neutropenia related to IC which would mandate either using mould-acting AF prophylaxis and/or systematic IFI biomarker monitoring (at least weekly)
4. Patient is willing and able to give informed consent for participation in the study

#### Exclusion criteria

Patients will be excluded from study entry if *any* of the following apply:

1. Previous proven or probable IFI (according to the EORTC/MSG criteria [13])
2. Contraindication to all potential prophylactic AF agents (i.e. cannot be prescribed any recognised anti-aspergillus agent as prophylaxis)
3. Planned chemotherapy using any regimen that mandates the use of systemic AF medication (i.e. venetoclax-based regimens)
4. Received >72 h of systemic mould-acting AF prophylaxis or therapy, or biomarker monitoring for IFI, prior to trial enrolment
5. Commenced the first cycle of chemotherapy >72 h prior to trial enrolment
6. Current diagnosis of *prolonged* (>72 h) of neutropenic fever
7. Pregnancy

### Informed consent {26a}

Once eligibility has been confirmed, written informed consent will be obtained from the patient by a suitably qualified and experienced local research nurse or clinician who has been authorised to do so by the Chief Investigator or recruiting site Principal Investigator, as detailed on the study Delegation of Authority and Signature Log for the study site.

Consent will be recorded via paper consent forms, which will be uploaded onto the secure web-based data collection interface Research Electronic Data Capture ‘REDCap’ once complete, or via participant electronic informed consent (e-consent) directly within the REDCap system.

### Additional consent provisions for collection and use of participant data and biological specimens {26b}

Alongside the main BioDriveAFS trial, there will be optional parallel studies available to trial participants at sites who are able and willing to be involved in these, and who have been invited to take part with the available local funding and approvals to do so. Participation in parallel studies will involve collecting a small number of additional samples (blood samples and/or skin/oral swabs, and breath samples) from participants who agree to this (both control and intervention arms). Further information on these studies, including objectives, is given in Additional File 1.

Patients will be able to participate in the main BioDriveAFS trial without consenting to the parallel studies, with no impact on their involvement in the main trial or on the quality of their routine clinical care. If the patient chooses to withdraw from the parallel studies, they can request that any stored samples be destroyed. Where sites are invited and agree to be involved in the parallel studies, a specific, tailored patient information leaflet (PIL) will be provided to patients who are approached about BioDriveAFS at that site, which will include details of the relevant additional optional studies. A specific, tailored consent form will be used at sites who are taking part in any of the parallel studies, which will include all main study consent statements, with statements for the additional parallel studies. This will allow consent for the main study and parallel studies to be taken at the same time to minimise any additional burden on patients and sites. The collection of samples will be aligned with routine blood tests/clinic visits wherever possible.

## Interventions

### Explanation for the choice of comparators {6b}

#### Control arm: prophylactic antifungals and standard of care approach

Patients will receive a prophylactic mould-acting AF in keeping with guidelines [14, 15]. As a minimum, prophylaxis should be given for the duration of chemotherapy-related neutropenia, at least until neutrophil recovery (>0.5 × 10^9^/L). Prophylaxis can be given either with each cycle of chemotherapy, or throughout and between subsequent cycles of IC, as per the usual SoC at the local study site. Some sites stop AF prophylaxis at neutrophil recovery between cycles of chemotherapy in routine care—this is acceptable if it is the usual practice at the study site. Monitoring (regular testing) with GM or BG while the patient is stable (i.e. does not have neutropenic fever (NF) or illness) will not be used in this arm. Patients with persistent NF lasting more than 96 h or with other symptoms suggestive of IFI will be investigated and managed according to existing local SoC (non-culture-based biomarker tests are allowed when used in a ‘reactive’ manner). Participants in this arm must receive prophylactic AF therapy with a recognised anti-aspergillus agent (posaconazole [used by 66% in the survey performed during trial design with key stakeholders and service providers], itraconazole [only when one of the other azoles cannot be used], isavuconazole, voriconazole, liposomal amphotericin B, or [when azoles cannot be used] anidulafungin, caspofungin or micafungin). Fluconazole cannot be used as the sole prophylactic agent.

### Intervention description {11a}

#### Intervention arm: biomarker-driven approach

Patients will have twice weekly blood tests for GM and BG from the start of IC until at least 7 days after neutrophil recovery with each cycle of IC as per usual local cut-off for neutropenia and clinical practice. Patients may spend a high proportion of their time as inpatients during this period, but during periods of lower risk (as deemed by the clinical care team), when the patient is being seen via outpatient clinics, testing can be reduced to once weekly or as often as the patient is attending (but no more than twice weekly) [i.e. patients do not require additional outpatient clinic appointments above what is the normal SoC to participate in this trial].

**Fig. 1:**
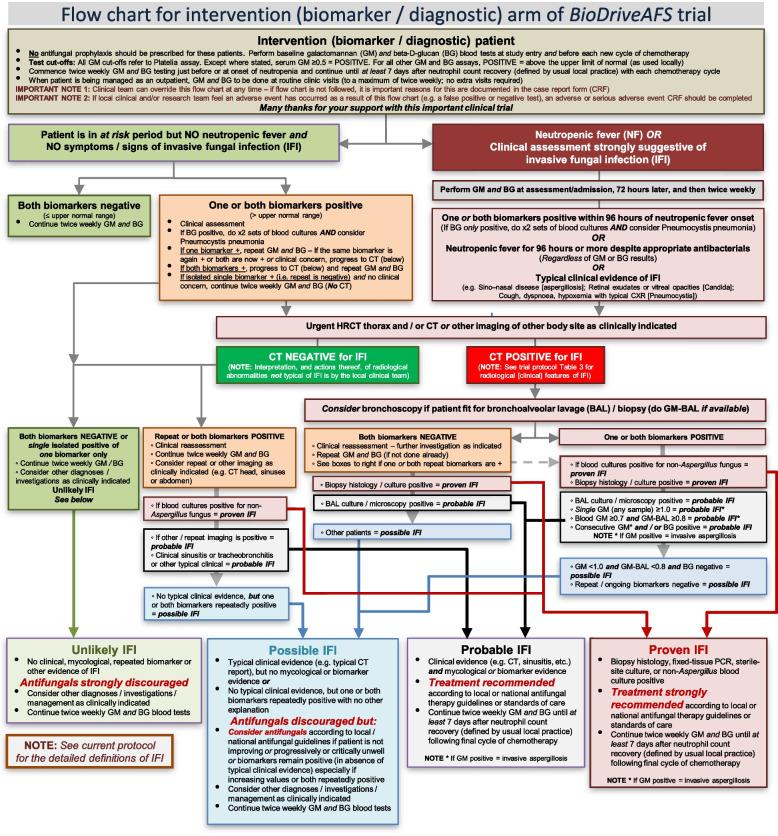
BioDriveAFS intervention arm flowchart

A clinical pathway approach (see intervention flow chart, Fig. 1), with integration of existing guidelines and definitions will guide the prevention, investigation and therapy of IFI [3, 13, 16, 17]. Whether symptoms are present or not, patients with two positive tests (either GM and BG both positive or GM or BG positive on consecutive occasions) will be recommended for an urgent high-resolution CT (HRCT) scan of the lungs (≤24 h or as soon as possible thereafter) and, if indicated, of other body sites. A bronchoscopy and AF therapy will be recommended if there are radiological features of IFI in line with guidance and, for centres with access to it, GM testing of bronchoalveolar lavage (BAL) fluid is recommended (though this is not mandated as part of the trial) [3, 16, 17]. If the patient meets the criteria, based on testing, for proven/probable IFI then targeted AF therapy according to site or national/international guidelines, at the discretion of the patient’s clinical team, will be recommended.

HRCT (± BAL), or other directed tests in line with guidance [3, 16], will also be recommended for patients with NF ≥ 96 h or other symptoms suggestive of IFI, but AF therapy will be discouraged if GM and BG remain negative in the absence of other evidence of IFI (proven/probable) [13]. In the survey performed during trial design with key stakeholders and service providers, the most used test for the investigation of IFI in an AML patient with prolonged NF was HRCT (75%) followed by GM (69%), BG (61%), BAL (58%) and then BAL GM (44%); these have all been incorporated into the trial care pathway.

In the event that a performed biomarker test fails due to technical or other reasons, the site team will repeat the test as soon as practically possible.

The clinical team will always retain the right to deviate from the pathway. When this occurs, it will need to be documented within the case report form (CRF) by site research teams. Additional biomarkers (e.g. Candida or Aspergillus PCR) cannot be used as regular IFI surveillance tools (as GM or BG are being used) but can be used ‘reactively’ at the clinical care team’s discretion during episodes of prolonged NF or when the patient exhibits other symptoms/signs of IFI (as is the case in the control/SoC arm).

### Criteria for discontinuing or modifying allocated interventions {11b}

BioDriveAFS is a pragmatic trial. Any modifications or changes to allocated interventions deemed clinically appropriate by a patient’s clinical care team will be recorded and reported in the trial CRFs. Participants can choose to withdraw from receiving the trial intervention in favour of SoC at any time.

### Strategies to improve adherence to interventions {11c}

The central trial team will be in regular contact with participating sites throughout the trial. Any potential issues raised by sites, including those that may impact adherence to the intervention (e.g. biomarker turnaround times) will be addressed appropriately. CRF return will be monitored on an ongoing basis, which will include auditing data on AFs and biomarkers.

### Relevant concomitant care permitted or prohibited during the trial {11d}

Additional clinical review and any further treatments will be determined by clinical need as per usual SoC.

### Provisions for post-trial care {30}

Following completion of their follow-up, participants will remain in the care of the treating clinicians as per usual clinical practice.

### Outcomes {12}

#### Co-primary outcome measures

1. AF exposure in the 12 months post-randomisation, defined as receipt of more than 72 h of therapeutic systemic AF.
2. Patient-reported EQ-5D-5L index utility value at 12 months post-randomisation. The EQ-5D-5L measures HRQoL in five dimensions: mobility, ability to self-care, ability to undertake usual activities, pain and discomfort, and anxiety and depression. EQ-5D-5L data will be collected via patient questionnaires by site research staff at baseline and then at 3, 6 and 12 months post-randomisation.

#### Secondary outcome measures, measured over 12 months

- Total AF exposure: total defined daily doses (DDD) and whole days of prophylactic and therapeutic AF use.
- Assessment of probable/proven IFI, which will be as per the consensus definitions of the Infectious Diseases Group of the EORTC/MSG [13]. The same definitions will be used for both arms of the trial (full definition provided below).
- Survival: all-cause mortality and IFI mortality.
- IFI treatment outcome, collected during the last follow-up assessment and categorised as treatment given and completed with no relapse; treatment given and completed, but with relapse; ongoing treatment; and IFI-related mortality.
- AF-associated adverse effects/events/complications using the adverse event (AE) reporting procedure and/or from relevant follow-up CRFs as appropriate.
- Resource use, collected to inform the economic evaluation. This will include hospital care health service use (e.g. length of hospital inpatient stay, readmissions and outpatient visits) and product costs. These data will be collected from hospital records and through patient questionnaires at baseline, and at 3, 6 and 12 months.
- Episodes of NF requiring hospital admission or outpatient management will be assessed using the standard European Society of Medical Oncology Clinical Practice Guidelines (ESMO CPG) definition [18].
- AF resistance in fungi (non-invasive and invasive) isolated from clinical specimens will be taken as part of routine care (i.e. additional samples will not be taken unless the patient has consented and the site is participating in additional sampling for storage/research).
- Desirability of Outcome Ranking (DOOR) [19], which is defined as hierarchical levels to be developed and confirmed following discussion with stakeholders, using Delphi methodology [20], and the trial Patient Advisory Group.

#### Definition of invasive fungal infection (IFI) in the BioDriveAFS trial

For a patient within the trial to be defined as having probable IFI they must have at least one clinical feature *plus* mycological evidence as detailed in Table 1 (all patients, by definition due to their diagnosis, will have at least one host factor), based on the definitions from the EORTC/MSG [13]. For the purposes of the proposed care pathway in the intervention (biomarker) arm, the cut-offs to trigger further investigation for IFI when the patient does not have NF are any value above the upper limit of the normal range for the test (GM or BG) being used, as defined by local definitions. For the purposes of diagnosis for the endpoints of the trial and where it states ‘probable/proven IFI’ in the proposed care pathway, the cut-offs in Table 1 will be used. The definitions for diagnosis of IFI will be the same for both arms of this trial. Patients who do not fit the EORTC/MSG definition of possible infection but who have persistently unexplained positive biomarkers (both GM or BG, or the same single biomarker consecutively), and are remaining to be or are progressively unwell, should be managed as a possible IFI in terms of considering therapeutic antifungals.

**Table 1:** Modified definitions for diagnosis of IFI based on EORTC/MSG criteria [13]

**Fungi**	**Proven**	**Probable**	**Possible**
		Requires 1 each of Host factor, Clinical Feature, and Mycological evidence	Requires 1 Host Factor, and 1 Clinical Feature ^a^ Requires 1 Host Factor, and persistently unexplained positive BG and/or GM
*Moulds*	• Sterile specimen demonstrating tissue invasion with hyphae or melanised yeast-like forms • Culture from sterile site, with associated clinical or radiological evidence of disease (excl. BAL, paranasal sinus, mastoid sinus, urine) • Growth from blood culture • Polymerase chain reaction (PCR) from fixed tissue specimen	*Host factors:* • Recent neutropenia (<0.5 × 10^9^/L) for >10 days • Active haematological malignancy • Previous allogenic stem cell or solid organ transplant • ≥0.3 mg/kg prednisolone equivalent for ≥3 weeks (past 60 days) • T-cell immunosuppression (last 90 days) • B-cell immunosuppression • Inherited severe immunodeficiency • Acute graft-versus-host disease (aGVHD) grade III/IV involving gut, lung, liver refractory to first-line steroids *Clinical features:* • Pulmonary Aspergillosis—dense, well-circumscribed lesion(s) +/− halo sign; air-crescent sign; cavity; or wedge-shaped and segmental or lobar consolidation • Other Pulmonary moulds—as above, or reverse-halo sign • Tracheobronchitis—tracheobronchial ulceration; nodule; pseudo-membrane; plaque; or eschar • Sino-nasal disease—acute localised pain, Nasal ulcer with black eschar, and extension across bony barriers • Central nervous system (CNS)—focal lesions or meningeal enhancement on magnetic resonance imaging (MRI)/ computed tomography (CT) *Mycological evidence:* • Microscopic detection of fungal elements in sputum, BAL, bronchial brushings or aspirate • Mould recovered by culture from sputum, BAL, bronchial brushings or aspirate • GM - Single serum or plasma, BAL fluid or CSF ≥1.0; or serum or plasma ≥0.7 AND BAL fluid ≥0.8 • BG ≥80 ng/L by Fungitell assay (^a^or above the positive cutoff value for other validated BG assays, as defined locally) in 2 consecutive serum samples with exclusion of other aetiology • Aspergillus PCR—blood, plasma or serum 2 consecutive positives; BAL fluid 2 positives; or blood, plasma or serum AND BAL fluid positive	
*Yeasts*	• Sterile-site specimen showing yeast cells • Culture from sterile-site specimen, with clinical or radiological evidence of infectious disease • Growth from blood culture • Positive cryptococcal antigen from blood or CSF • PCR from fixed tissue specimen	Candida *Host factors:* • Recent neutropenia (<0.5 × 10^9^/L) for >10 days • Active haematological malignancy • Previous allogenic stem cell or solid organ transplant • ≥0.3 mg/kg prednisolone equivalent for ≥3 weeks (past 60 days) • T-cell immunosuppression (last 90 days) • Inherited severe immunodeficiency • aGVHD grade III/IV involving gut, lung, liver refractory to first-line steroids *Clinical features:* • Candidaemia in the past 2 weeks with 1 of: • Small target-like abscesses in liver, spleen or brain, or meningeal enhancement • Progressive retinal exudates or vitreal opacities *Mycological evidence:* • BG ≥80 ng/L by Fungitell assay (^a^or above the positive cutoff value for other validated BG assays, as defined locally) in 2 consecutive serum samples with exclusion of other aetiology • Positive T2Candida assay Cryptococcus *Host factors:* (may occur in phenotypically normal patients) • Human immunodeficiency virus (HIV) • Stem cell or solid organ transplant • Haematological malignancy • Antibody deficiency • Immunosuppressive therapy • End-stage liver or renal disease • Idiopathic CD4 lymphocytopenia *Clinical features:* • Meningeal inflammation or consistent radiological lesion *Mycological:* • Recovery of Cryptococcus from any non-sterile site	
*PJP* (PCP)	• Detection of organism microscopically in tissue, BAL or expectorating sputum	*Host factors:* • CD4 count <200 cells/mm^3^ • Medication causing T-cell dysfunction • ≥0.3 mg/kg prednisolone equivalent ≥ 2 weeks (past 60 days) • Solid organ transplant *Clinical features:* • Consistent radiographic features • Respiratory symptoms with cough, dyspnoea and hypoxemia accompanying radiographic abnormalities *Mycological:* • BG ≥ 80 ng/L by Fungitell assay (^a^or above the positive cutoff value for other validated BG assays, as defined locally) in 2 consecutive serum samples with exclusion of other aetiology • Detection of Pneumocystis pneumonia (PCP) Deoxyribonucleic acid (DNA) by PCR in a respiratory tract specimen	
Endemic Mycoses	• Histopathology or microscopy or specimens obtained by an affected site showing distinctive forms of the fungus • Recovery of fungus from an affected site • Growth from blood culture	*Host factors:* • Can occur in any patient *Clinical features:* • Geographical or occupational exposure with compatible clinical illness *Mycological evidence:* • Histoplasma or Blastomyces antigen in urine, serum or fluid • Coccidioides antibody in cerebrospinal fluid (CSF), or 2-fold rise in 2 consecutive serum samples	

^a^Indicates deviations from the EORTC/MSG criteria

### Participant timeline {13}

**Fig. 2:**
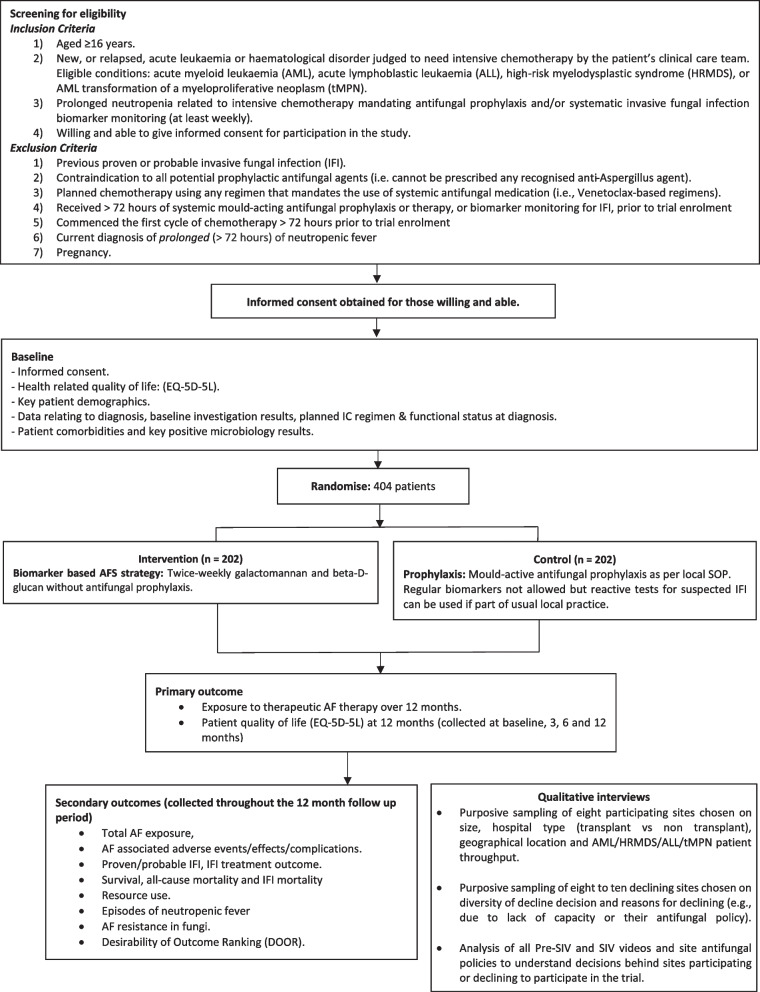
BioDriveAFS participant timeline

Participant timeline is shown in Fig. 2.

### Sample size {14}

This trial has two co-primary endpoints (AF exposure over 12 months and EQ-5D-5L at 12 months) and is powered such that success must be shown for both outcomes for the intervention to be deemed beneficial (see Table 2); therefore, no adjustment for multiple comparisons is required. The comparison of AF use between the two groups is based on showing superiority, while the comparison of EQ-5D-5L index values is based on non-inferiority. The sample size is calculated at 404 patients, as follows.

**Table 2:** Criteria for decisions about trial effectiveness based on co-primary endpoints

	**Reduced systemic AF use in intervention arm (statistical superiority)**	**Equivalent or more systemic AF use in intervention arm**
**HRQoL non-inferior**	*Effective*	Ineffective
**HRQoL not non-inferior for intervention**	Ineffective	Ineffective

#### Sample size for antifungal therapy use

Based on published AF use, an estimated 30% of acute leukaemia patients will receive ≥3 days of therapeutic systemic AF during IC with AF prophylaxis/SoC [21]. Studies of biomarker-led approaches have shown reductions in AF use of >50% [22, 23]. To identify a reduction in this binary outcome from 30 to 15% of patients, with 90% power and two-sided statistical significance of 5%, and allowing for 20% attrition, requires 404 patients.

#### Sample size for health-related quality of life (EQ-5D-5L)

Pickard et al. estimated the minimal clinically important difference for the EQ-5D-3L UK-utility scores in cancer patients (all cancers) to be between 0.09 and 0.12 [24]. McClure and colleagues found a difference of 0.063 for the EQ-5D-5L using simulated data for a general population [25]. Accounting for 15% attrition (participants known to be alive but lost to follow-up; participants who die can be given a score of 0 for any assessment time point following their date of death), a sample size of 404 will be sufficient to assess the hypothesis that the intervention is non-inferior to control, based on a non-inferiority margin of 0.07, SD 0.20 [26], 90% power and a 95% two-sided confidence interval.

#### Sample size for proven/probable fungal infection (key secondary clinical outcome)

This sample size will also provide adequate power for the key secondary outcome of proven/probable IFI, to show that the intervention does not increase this outcome by more than 5% provided the proportion in the control group is no more than 2% [21], allowing for 20% attrition.

### Recruitment {15}

Patients will be provided with a paper or electronic PIL. For those unable to speak English, either a translator or language line will be used depending on local availability. Patients will have the opportunity to ask questions of the recruiting research team (i.e. research clinician or nurse) and given as much time as they need to decide if they would like to take part before completing consent processes, within the time constraints of clinical decisions associated with commencing their treatment.

To ensure diversity of participation, data will be collected at screening/randomisation about patient characteristics that could potentially impact trial endpoints such as age, postcode (to estimate index of multiple deprivation score (IMD), with only the first half of the postcode collected from non-randomised patients), ethnicity and sex, which will be monitored by the Trial Management Group (TMG) and the independent Trial Steering Committee (TSC).

The central study team will work closely with the treating clinicians and local research teams at each participating site via engagement, training and networking to optimise the local screening and recruitment processes, initially at the point of site set-up and initiation. Thereafter, there will be various opportunities to adapt and optimise this further; including at planned site training events, through real-time site networking, based on advice from patient/public involvement meetings and learnings from the pilot phase process evaluation work.

The PIL will be developed in close collaboration with the Patient Advisory Group (PAG) to ensure that this is accessible to this patient group and presents all relevant information appropriately. All information required by the UK Health Research Authority (HRA) will be included. Throughout the whole study, screening logs will be kept at each site to determine the number of patients assessed for eligibility and reasons for any exclusion, including any given for patients declining involvement, to help to inform where action may be necessary to maximise recruitment.

## Assignment of interventions: allocation

### Sequence generation {16a}, Concealment mechanism {16b} and Implementation {16c}

The allocation sequence will be generated in Stata v17, by a statistician at York Trials Unit (YTU) not involved in the recruitment of participants using block randomisation stratified by site, with randomly permuted block sizes.

Once eligibility has been confirmed, consent has been obtained, and baseline data collected, participants will be randomised 1:1 (Intervention : Control) by local site staff using REDCap.

## Assignment of interventions: blinding

### Who will be blinded {17a}

Due to the nature of the intervention, this is an unblinded trial, and treating clinicians will be informed of the allocation via the online secure REDCap system and will then tell the patient. Therefore, an unblinding procedure is not required for this study.

### Procedure for unblinding if needed {17b}

Not applicable as no blinding will be used in this trial.

## Data collection and management

### Plans for assessment and collection of outcomes {18a}

**Fig. 3:**
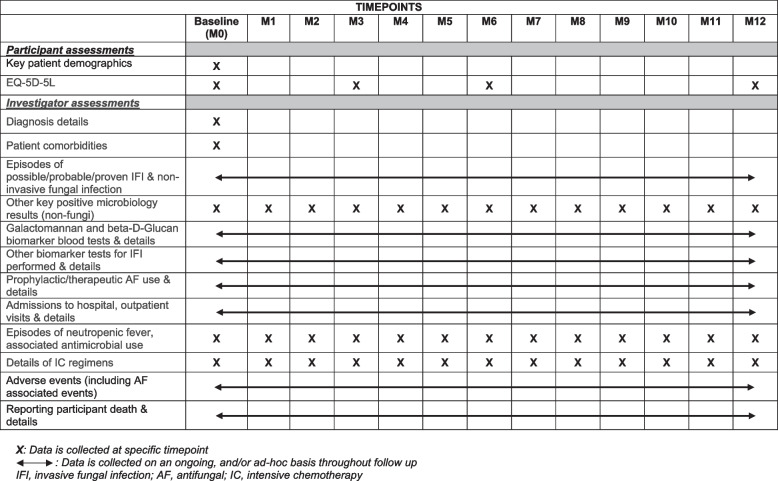
BioDriveAFS data collection assessment schedule

Data will be collected using bespoke CRFs completed electronically via REDCap, or collected on paper CRFs returned via post to YTU with data then manually entered into REDCap. An overview of the data collection assessment schedule is given in Fig. 3.

Investigator-completed hospital CRFs will only be completed by personnel authorised to do so by the Principal Investigator, as recorded on the trial-specific delegation log for each hospital site. Investigators will be trained in data completion by the central study team prior to commencing work on the trial and data entry and refresher training will be made available whenever required. A trial manual detailing all trial processes will be provided to participating sites. Investigator-completed data can be submitted at any stage during the participant’s follow-up and reminders will be sent to research staff at sites to do this.

### Plans to promote participant retention and complete follow-up {18b}

To minimise attrition, several methods will be used to keep in contact with participants. Multiple options are available for questionnaire completion depending on participant preference and to allow alternative options where there is no response initially: online via a REDCap link to their email where an email address is provided, a REDCap link sent via SMS (using the secure UK-based text message gateway software ‘IntelliSMS’—https://www.intellisoftware.co.uk) if the patient provides a mobile phone number, postal completion, or completion over the phone with the study team at YTU. The participant follow-up questionnaires at 3, 6 and 12 months are short, only containing the primary outcome (EQ-5D-5L).

Pre-notification emails will be sent to participants (where an email address has been provided) 1 week before the follow-up questionnaires are due, to help prime them to complete this when the email is sent containing the REDCap link [27]. Automated reminder emails are also sent 1 and 2 weeks after the due date. Where no response is received to email questionnaires, participants will be given the option to complete over the phone or via post if preferred.

Participants will be given an unconditional £10 voucher with each follow-up questionnaire (at 3, 6 and 12 months). Due to the nature of their illness, patients may be very unwell at certain time points. Research teams at hospital sites will be contacted by the trial team to ensure this is taken into account for questionnaire completion, for example when completing these over the phone with patients, to ensure they are contacted at appropriate times.

Newsletters will also be sent to participants throughout the trial to keep them informed and engaged with the study [28]. Patient/public contributors will be heavily involved throughout the trial design, recruitment and follow-up periods to ensure methods used are the most appropriate for this patient group.

Patients are free to withdraw from data collection at any point without any compromise or change in their clinical care. It will be possible for them to withdraw only from one aspect of data collection if needed. For example, they could continue with participant questionnaire completion only, or continue with only data collection from their hospital records with no participant-completed data. This should minimise the need for patients to fully withdraw and enable maximum data to be collected.

### Data management {19}

#### Data entry and storage

The data collected by sites will be entered onto the secure web-based REDCap interface. Data will be held securely on a cloud-hosted REDCap server. Access to the study interface will be restricted to named authorised individuals granted user rights by a REDCap administrator at YTU.

#### Data protection

Data will be processed in accordance with the General Data Protection Regulations (GDPR) and the Data Protection Act 2018.

#### Management of qualitative data

This relates to qualitative data collected as part of the Process Evaluation (see ‘Mixed methods process evaluation’ section). All qualitative data will be analysed and stored at the University of York (UoY), UK. Audio data will be removed from recording devices as soon as is practicable and will be transferred, stored on and accessed via secure, password-protected servers at UoY. Audio files will be transcribed in house by a trained typist (an administrator at UoY). A confidentiality and data security agreement is in place and only research team members can access data. Separate verbal consent audio recordings will be stored for 5 years, and then deleted. Interview audio recordings will be deleted as soon as possible following transcription. Interview transcripts and any paper data will be stored for a period of 10 years, when paper data, confidential waste and electronic data no longer required for analysis will be disposed of/deleted.

### Confidentiality {27}

To maintain anonymity and confidentiality, patients will be assigned a Unique Trial Number which will be used to identify participant data. All study data used for analysis will be pseudonymised using only the Trial Number to identify the patient, and patients will not be identifiable in any reports or publications. All data will be processed and stored in accordance with the GDPR and the Data Protection Act 2018. All records will be stored in secure locked locations, with consent forms stored securely on password-protected servers and/or in secure locked cabinets with authorised access only. Only the study team, the Sponsor, the NHS Trust or regulatory authorities will review clinical information where it is relevant to the patient taking part in the research, agreed by the patient at the time of consent.

### Plans for collection, laboratory evaluation and storage of biological specimens for genetic or molecular analysis in this trial/future use {33}

Clinical samples, for example the serum biomarker tests as part of the intervention arm, will be collected, handled and analysed according to routine NHS procedures. Optional parallel studies will take place at some sites involving collection and analysis of additional biological samples, which are described in further detail in Additional File 1.

## Statistical methods

### Statistical methods for primary and secondary outcomes {20a}

Full analyses will be detailed in a Statistical Analysis Plan (SAP), which will be finalised prior to the end of data collection, and reviewed and approved by the TMG and independent oversight committees (Data Monitoring and Ethics Committee (DMEC) and TSC). Analyses will be carried out using two-sided statistical tests at the 5% significance level under the principles of intention-to-treat. The trial will be reported according to the CONSORT guidelines for clinical trials and participant flow will be presented in a CONSORT diagram [29].

#### Main trial analysis

Baseline data will be summarised descriptively by trial arm, both as randomised and as included in the primary analyses. No formal statistical testing will be conducted on baseline data.

For intervention participants, the following will be summarised: the number and frequency of their blood tests for GM/BG from the start of IC until neutrophil recovery after the final IC; the number who undergo a HRCT scan following one or two positive tests but without symptoms; the number who undergo a bronchoscopy and GM BAL; and those prescribed systemic AF therapy among those with/without features of proven/probable IFI. For patients with NF ≥96 h or other symptoms suggestive of IFI, the number who undergo a HRCT (± GM BAL) or other directed tests and/or are prescribed systemic AF therapy will be summarised, stratified by whether or not their GM/BG remain negative. The same measures will be assessed in the comparator group to assess for contamination. It would be expected that the use of GM/BG during periods of clinical stability (i.e. no neutropenia and/or fever and/or IFI symptoms) will to be zero or very low in the control group. The use, defined daily doses, and full days of therapy of prophylactic and therapeutic systemic AF for all participants over the course of the trial will be summarised.

The co-primary outcome of AF use, as a binary outcome, will be analysed via a mixed-effects logistic regression model, adjusting for pertinent participant-level covariates as fixed effects and site as a random effect. EQ-5D-5L index values will be compared between the two groups using a covariance pattern linear mixed model incorporating all post-randomisation assessment points adjusting for baseline value, other pertinent baseline covariates, time and an interaction between treatment group and time as fixed effects. Participant and site will be specified as random effects. The adjusted mean difference in EQ-5D-5L score over the whole 12 months and at each time point will be calculated with its 95% confidence interval; the treatment effect at 12 months will be the primary endpoint, while the other differences will serve as secondary investigations.

Secondary outcomes will be analysed using appropriate regression techniques; for example, logistic regression for probable/proven IFI, and the presence of AF associated adverse events; Cox Proportional Hazards regression for survival outcomes (time to all cause and IFI mortality); a proportional odds logistic model for the DOOR outcome; and Poisson regression for count data of number of episodes of NF requiring hospital admission or outpatient management.

### Interim analyses {21b}

No formal interim analyses will be performed.

Relevant data from the internal pilot trial (first 9 months of recruitment) will be assessed against predefined progression criteria (Table 3) by the TSC, DMEC, PAG and the NIHR prior to progression to the main trial to help determine whether continuation is warranted. The NIHR will make the final decision as to whether the trial continues or is terminated.

**Table 3:** Internal, 9-month pilot phase progression criteria

	**Red**	**Amber**	**Green**
**Average number of patients randomised per site per month**	<0.3	0.3–0.59	≥0.6
**Number of sites opened**	<9	9–12	≥13
**Adherence with intervention pathway**^a^	<50%	50–74%	≥75
**Collection of valid EQ-5D-5L at month 3 assessment**	<70%	70–89%	≥90%

^a^for intervention participants only, defined as having 3 or more blood tests for GM and BG in the 4 weeks from randomisation

### Methods for additional analyses (e.g. subgroup analyses) {20b}

Any additional analyses to those detailed in this protocol will be detailed in the SAP, which will be finalised prior to the end of data collection, and reviewed and approved by the TMG, DMEC and TSC.

#### Health economic evaluation analyses

The objective of the economic evaluation analysis is to assess the cost-effectiveness of the biomarker-led diagnostic strategy in preventing and managing IFI in patient with AML/ALL/HRMDS/tMPN, as compared to prophylactic AF/SoC. The evaluation, conducted from the perspective of the NHS and personal social service (PSS), aims to analyse the health benefits and associated costs of both interventions.

Cost calculation will employ a bottom-up methodology encompassing resources necessary for intervention delivery and individual health and social service utilisation over the study period. Data on resource use will be collected from participating sites though tailored CRFs completed by health care staff (i.e. costs associated with prophylaxis/empiric AF, AF related AEs, biomarker implementation and testing, length of stay, readmissions and follow-up visits related to infections). Collected resource use information will be multiplied by unit costs obtained from authoritative sources including the National Cost Collection by NHS England [30] the Unit of pf Health and Social Care report by Personal Social Services Research Unit (PSSRU) [31] and other appropriate national sources.

Health outcomes will be expressed in terms of the quality-adjusted life year (QALY), utilising the EQ-5D-5L instrument to measure the impact on both quantity and quality of life. Individual-level responses to EQ-5D-5L will be used to calculate utility scores based on UK population value set, and an area under the curve approach will be used to calculated QALYs [32]. To handle the missingness, multiple imputation with chained equations will be employed to address anticipated missing data for resource use and utility measures, imputing costs and utility measures based on patient characteristics and baseline costs and QALYs [33, 34].

The primary economic analysis will be a within-trial cost-utility analysis conducted at the end of trial (12-month follow-up) to evaluate the short-term cost-effectiveness. Mean total costs and QALYs will be compared between two interventions using regression models, controlling for baseline characteristics, such as baseline utility [35]. These models will not only consider the distribution of the cost and QALY estimates but also the correlation between them [36]. To handle the uncertainty, a non-parametric bootstrap will be used to produce confidence intervals around the incremental cost, incremental QALY and incremental cost-effectiveness ratio (ICER), as the regression residuals are likely to be skewed [37]. Bootstrapped results will be graphically presented in the conventional form of a cost-effectiveness plane (CE-plane) and cost-effectiveness acceptability curve (CEAC), with the calculated ICER assessed against the NICE willingness-to-pay (WTP) threshold of £20,000 to £30,000 per QALY gained to determine the short-term cost-effectiveness of the biomarker-led diagnostic.

For long-term effects, a decision-analytic model may be developed to project costs and QALYs over the patient’s lifetime. The model structure will be informed by existing literature and expert consultations, while parameters will be sourced from previous modelling studies and the best available evidence from the literature. A 3.5% annual discount rate will be applied to both costs and QALY predictions. Same to the short-term within-trial analysis, the projected results will be plotted, and the ICER will be calculated against the WTP threshold to assess the long-term cost-effectiveness of biomarker-led diagnostic strategy.

This study will follow the National Institute for Health and Care Excellence (NICE) Guide to the Methods of Technology Appraisal [38], and a detailed health economics analysis plan (HEAP) will be drawn up in advance of the analysis and approved by the trial DMEC and TSC.

A within trial analysis with total costs and QALYs will be presented for both trial groups. This analysis will be conducted using regression methods and will assess the short-term effect on patients’ health and costs to the NHS of the interventions in the trial.

However, it is unlikely to provide all the evidence relevant to the decision on whether a biomarker-led strategy represents a cost-effective option for the NHS. Hence, a decision-analytic model will be developed to extrapolate the effect on lifetime costs and QALYs combining the best available evidence. A state-transition model will be used in this analysis. State-transition models use a series of health states which demark important changes to prognosis, costs or quality of life. Parameter estimates, including HRQoL associated with long-term consequences of infections, will be sourced from primary data sources, previous modelling studies and the best available evidence from the literature. Systematic searches will be conducted to update the most comprehensive evidence in this area. A 3.5% annual discount rate will be applied for costs and outcomes.

## Mixed methods process evaluation

### Aims

The overall aim of the process evaluation is to robustly evaluate how the intervention is delivered during the internal pilot and main trial via the collection of both qualitative and quantitative data. The specific aims of the process evaluation are to:

1. Understand the contexts/settings in which the intervention works better, and why (qualitative);
2. Explore implementation barriers/facilitators to inform post-trial implementation (qualitative);
3. Assess fidelity to the clinical pathway (quantitative and qualitative).

#### Quantitative data collection—assessment of fidelity to the clinical pathway

Quantitative measurement will focus on the core principles of adherence, defined as:

- *Content:* did the clinical team deliver the intervention as designed by the research team?
- *Frequency and duration:* did the clinical team deliver the intervention as often and as long as planned, based on pre-specified targets?
- *Coverage:* was the intervention delivered to all eligible participants?

The *assessment criteria* will include exploring:

- Whether participants undergo GM/BG testing as per the care pathway and if not, why not? (information collected will include frequency of testing and duration).
- Modifications/adaptations to the care pathway and the reasons behind this.
- Whether patients received AF therapy diverging from the care pathway.

##### Sample:

Data will be collected for every intervention patient enrolled in both the pilot and main trial (about 200 patients), across all sites.

##### Procedure:

**Fig. 4:**
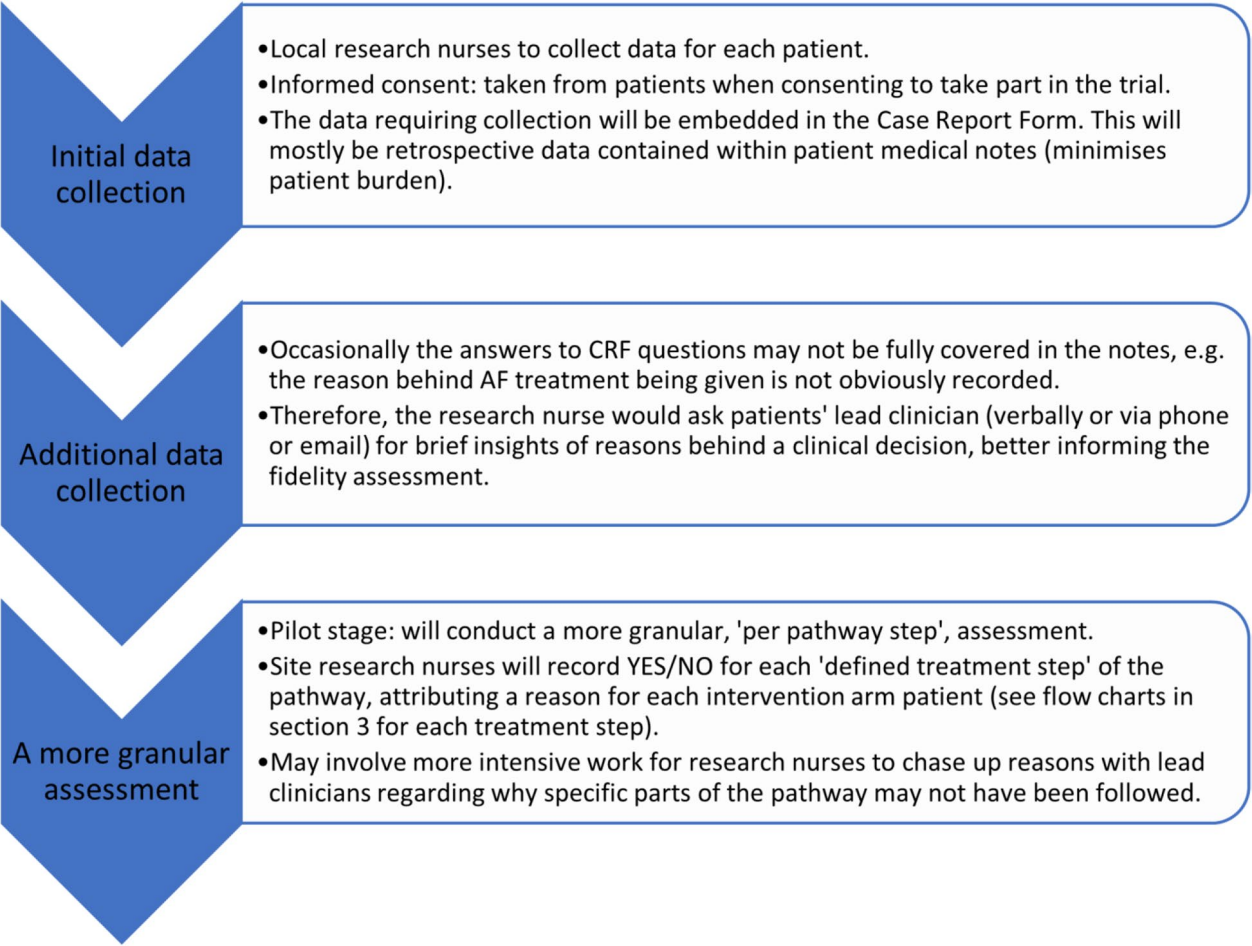
Quantitative fidelity assessment procedure

Figure 4 shows quantitative fidelity assessment procedure.

##### Fidelity scoring:

An intervention fidelity scoring matrix will be developed [39]. Towards the end of the study an aggregate score will be produced from the three fidelity domains (content, frequency and duration, coverage). Adherence will be categorised on a scale of 0–3 (0 being no adherence, and 3 for full adherence) for each site with interviews conducted with the lead clinician at 10 sites to understand site level fidelity.

#### Qualitative data collection—understanding context and exploring implementation

There are five components to the qualitative work with a primary goal to understand ‘what works, for whom, when and why?’ It will capture contextual site factors that may shape intervention implementation and delivery, alongside levers behind accepting or declining to take part in the trial.

##### Patient interviews:

Phone or video interviews lasting approximately 40 to 60 min will be conducted with a purposive sample of 40 unique intervention arm participants overall (20 patients at two different timepoints) after hospital discharge. Participants will be sampled on age, gender, ethnicity and length of hospital stay.

The aim of the patient interviews is to understand patients’ perceptions of the intervention by exploring their experience of hospital treatment and knowledge of the intervention. The topic guide will be developed with PPI input and will have questions on areas such as inpatient experience and how the intervention was delivered.

In collaboration with the process evaluation researcher, Research Nurses (RNs) will identify participants who might be interested in taking part. Permission will be sought to forward their contact details to a researcher at the UoY. Either an information sheet and consent form will be given to patients for consideration while in hospital by RNs (where they feel it is appropriate) or the researcher will provide these documents (post or email) after first contacting the patient via phone to gauge their interest in taking part. Once they agree to participate and an interview is set up, verbal consent will be obtained during the interview via audio recording. They will be reassured that involvement is entirely voluntary, the interview can be stopped at any time and they have a right to withdraw without any effect on their medical care.

##### Healthcare staff interviews:

A mixture of face-to-face, phone and video interviews lasting around 30 min will be conducted with approximately five healthcare professionals per site who are key implementers of the intervention and those who provide clinical care for patients (e.g. haematologists, infection doctors, pharmacists, nurses, allied health professionals and lead RNs who recruit patients to the trial). In total, staff from eight sites will be interviewed, with a total of 40 participants interviewed during the pilot phase and 40 participants interviewed towards the end of the trial. There will be a mixture of individual or focus group interviews, depending on preferences.

Healthcare professionals will be asked to talk in a non-identifying manner about an intervention and a control arm patient, to ground focus. The topic guide will be based on core constructs of Normalisation Process Theory [40] and will focus on areas such as practicalities of the intervention, problems and successes, systems/relationships/site set-up and any changes in practice occurring with control group patients.

A researcher from YTU will invite staff to take part in an interview/focus group. The initial approach will be via email or a short verbal description of what is involved. RNs will likely identify healthcare staff for interviews. If there is interest in being involved, an information sheet will be provided with opportunities to ask questions. Those who subsequently agree to participate will be emailed a consent form. Audio recorded verbal consent will be taken.

##### Lead clinician interviews:

Brief structured one-off telephone interviews will be conducted with lead clinicians from the five least and five highest adherent sites. The approach and consent process will be the same as for healthcare staff.

Questions will be based on the moderating factors developed via staff and patient interviews. Lead clinician’s thoughts as to why the intervention may have succeeded well or less well at their site will be encouraged. Lead clinicians will also be asked why their site agreed to participate in the trial.

##### Declining site interviews:

Brief semi-structured one-off telephone interviews will be conducted with clinicians at 8–10 sites that have declined to take part in the BioDriveAFS trial. It is anticipated these interviews will last approximately 20 min. The approach and consent process will be the same as described above.

These interviews will explore and provide more nuanced understandings of why sites decline.

##### Site initiation ‘visit’ (SIV) video analysis:

As part of the pilot phase, YTU staff will conduct SIVs when a site indicates it is ready to go ahead with the trial. This will also include a preliminary meeting between YTU and site teams (pre-SIV) to discuss clinical aspects of the trial. These video calls will be recorded as standard practice and analysed as part of the process evaluation. This will help provide an understanding of the levers of accepting and declining trial participation as well as the context of practices within sites where the trial would be situated.

Each clinical team member appearing in the recording will be emailed asking for their consent to include their contribution and conversation in the analysis (with an explanation of the approach and a ‘further information’ sheet). If an individual declines, their contribution will not be included in the analysis. If no email response is received, two further emails will be sent with an option to opt out. If there is still no response, the YTU research team will proceed to include their contribution (they will be clearly made aware of this in emails). There will be no reference to individual patients or individual care provision in recordings.

#### Other data

A summary of other data collection methods, samples and timepoints for the process evaluation can be found in Additional File 2.

#### Process evaluation analysis plan

Analysis of *quantitative data* will include basic descriptive statistical analysis. After the pilot stage of the trial, analysis will pay attention to interim levels of adherence and differences between sites. Towards the end of the trial, analysis of fidelity data from the main trial will be undertaken. A fidelity scoring matrix will also be used.

Analysis of *qualitative data* will include a rapid descriptive thematic analysis to generate headline themes emerging during the trial. Towards the end of the recruitment period, there will be a mixture of deductive analysis and inductive descriptive analysis to explore intervention implementation and fidelity. Towards the end of the trial, deductive analysis will be undertaken to analyse fidelity as well as framework analysis of responses.

Finally, after the pilot stage, there will be a *mixed methods integration* of qualitative and quantitative data to refine the treatment pathway/clinician training to improve adherence moving forward into the main trial.

Additional File 2 shows further in-depth details regarding analysis methods, frameworks and outcomes.

### Methods in analysis to handle protocol non-adherence and any statistical methods to handle missing data {20c}

Complier Average Causal Effect sensitivity analyses for the primary outcomes will be conducted to account for non-compliance with the intervention and contamination, which will consider the number and frequency of GM/BG tests undertaken for participants over the relevant follow-up period.

### Plans to give access to the full protocol, participant-level data and statistical code {31c}

The full protocol is available via the Funder website: https://www.fundingawards.nihr.ac.uk/award/NIHR132674

In principle, once analysis and all intended outputs are complete, anonymised data will be made available for meta-analysis and where requested by other authorised researchers and journals for publication purposes. Requests for access to data or documentation will be reviewed by the Chief Investigators and study Sponsor.

## Oversight and monitoring

### Composition of the coordinating centre and trial steering committee {5d}

YTU will lead on overall trial management and governance in close collaboration with the Co-Chief investigators and co-applicants. YTU has an established track record of running large clinical trials and will be responsible for delivering the trial with quality assured trial processes. YTU will communicate regularly with trial sites and monitor trial activities to ensure compliance with Good Clinical Practice (GCP). Key members from YTU will be part of the TMG, including the trial statisticians, trial manager, trial coordinators, health economist and qualitative researcher.

The TMG will meet approximately bimonthly via videoconference/teleconference or in person.

The TSC is independent and established to provide overall independent oversight for BioDriveAFS on behalf of the Sponsor and Project Funder and to ensure that the project is conducted to the rigorous standards set out in the Department of Health’s Research Governance Framework for Health and Social Care and the Guidelines for GCP. The committee comprises an independent academic haematologist, a pharmacist specialising in antimicrobial stewardship, an infectious diseases physician, a biostatistician, a health economist, an academic researcher with expertise in process evaluation work and a patient/public contributor. The TSC will meet routinely during the trial to monitor the progress of the trial and provide independent advice. A Sponsor representative will also be invited to attend TSC meetings.

### Composition of the data monitoring committee, its role and reporting structure {21a}

The BioDriveAFS DMEC is independent of the study sponsor and comprises independent clinicians, a statistician and a pharmacist. All DMEC members have signed a DMEC charter and confirmed they have no competing interests. This is stored in the trial master file at YTU.

The DMEC will meet annually (or more frequently if the committee requests) to provide project oversight to the trial. This will include monitoring safety and efficacy data, and quality and compliance data, while ensuring the protocol is accurately followed, and the study is GCP compliant. The committee will recommend whether there are any ethical or safety reasons why the trial should not continue, and report these in writing to the TSC. Independent members of the DMEC committee will be allowed to see unblinded data on request.

#### Patient and public involvement (PPI)

The BioDriveAFS Trial protocol was developed with input from the Patient Advisory Group (PAG), including primary and secondary outcomes (informing the choice of the EQ-5D-5L quality of life assessment as a co-primary outcome), study assessment schedule, inclusion and exclusion criteria, outcome assessment tools and ways to support diversity and inclusivity in the trial.

The PAG will meet regularly throughout the study and will continue to work with the study team to optimise recruitment, retention and dissemination of findings through activities such as the co-development of study documents and communication tools. Their contributions will help to ensure that documentation and dissemination is engaging and accessible for patients, their carers and the public.

PPI contributors will be part of the relevant trial committees, with two PPI members on the TMG, and one member on the TSC.

### Adverse event reporting and harms {22}

The BioDriveAFS trial will comprise adult patients with acute leukaemias undergoing IC. Prolonged hospital inpatient admission and complex clinical events, common in this patient group, will be captured within trial CRFs and will not necessarily require AE reporting. For the purposes of the BioDriveAFS trial, AEs are defined as any untoward medical occurrence (i.e. any unfavourable and unintended sign, symptom or disease) in a trial participant that logically could or is likely to have a causal relationship with the intervention (i.e. intervention pathway biomarker/diagnostic tests and associated treatments thereof). This could include AEs as a result of, for example, interventions (tests or treatments) that occur because of a false positive biomarker result or AEs due to a lack of an intervention because of a false negative biomarker result.

The following events will not need to be reported routinely as an AE for this trial unless the criteria above or serious adverse event (SAE) criteria are fulfilled:

- Respiratory infection or failure, including mechanical ventilation and acute lung injury
- Hepatic infection or failure
- Renal infection or failure, including the need for renal replacement therapy
- Haematological/coagulation failure, including anaemia, leucopenia, thrombocytopaenia or pancytopaenia
- Neurological infection or failure
- Unscheduled care escalation
- Infection relapse/recurrence requiring further antimicrobials
- Super- or secondary infection defined as a new infection at a different body site
- Suspected antimicrobial adverse reactions/events
- Progression of the underlying haematological disease or non-response to systemic antineoplastic chemotherapy
- AEs related to the antineoplastic chemotherapy

Although the above will not require expedited reporting as an AE on the study, key complications will be captured in other routine follow-up CRFs. For example, details of fungal infections, and key bacterial and viral infections (including NF) will be captured on a monthly basis. Attendance at and admission to hospital for reasons relating to the management of a participant’s leukaemia will be captured.

For the BioDriveAFS trial, SAEs are defined as events resulting in (i) persistent or significant disability or incapacity or (ii) a congenital anomaly or birth defect.

All SAEs should be reported to YTU within 24 h of the investigator becoming aware of the event. Once received, causality and expectedness will be confirmed by one of the Co-Chief Investigators or a medical co-applicant or TSC member not acting as a site Principal Investigator (PI). Any change of condition or other follow-up information should be sent as soon as it is available or at least within 24 h of the information becoming available. Events will be followed up until the event has resolved or a final outcome has been reached.

AEs that are deemed to be unexpected and related to the trial will be notified to the REC and sponsor within 15 days. All such events will be reported to the TSC and DMEC at their next meetings.

### Frequency and plans for auditing trial conduct {23}

The study will be conducted in accordance with the current approved protocol, ICH GCP, relevant regulations, standard operating and trial-specific procedures.

Regular central monitoring will be performed according to ICH GCP and the BioDriveAFS monitoring plan. The BioDriveAFS monitoring plan which will be agreed by the Sponsor, TMG, TSC and Co-Chief Investigators. Data will be evaluated for compliance with the protocol and GCP and the applicable regulatory requirements.

### Plans for communicating important protocol amendments to relevant parties (e.g. trial participants, ethical committees) {25}

Substantial protocol changes will firstly be agreed with the Funding Body, Sponsor, TSC, DMEC and TMG. Agreement for minor protocol changes will be sought from the TMG and Sponsor. Amendments will then be made to the required documentation, and the HRA amendment tool completed to confirm the category of the amendment. Once Sponsor authorisation has been confirmed, YTU will submit via IRAS and, where necessary, obtain approval from the Research Ethics Committee (REC), HRA and host institution(s) for approval of all substantial amendments to the original approved documents. Once approvals are received, the new documents/versions will be shared with sites and the study version control log will be updated for sites to check they are using only the most recent versions of trial documents.

For any amendments to trial eligibility criteria, the ISRCTN registry will also be updated. Trial participants will be written to, if necessary, to explain any changes.

### Dissemination plans {31a}

Through the planned methods and outputs, the study is expected to play a key role in enhancing the evidence base on the effectiveness of a biomarker-based antifungal stewardship strategy vs a prophylactic AF strategy in reducing AF therapy use in patients with AL undergoing IC. The economic analyses will help identify the most efficient and responsible (in terms of AFS) provision of future care and thus savings and benefits to the NHS and society.

Results from this study will be written up and submitted to peer-reviewed journals. Several dissemination channels will be used to ensure patients and the public are also informed of the study results. Engagement will continue to take place throughout the trial and beyond with key stakeholders, partners and collaborators as part of the dissemination strategy. These include relevant charities and patient organisations, relevant NIHR Applied Research Collaboratives, key opinion leaders (e.g. in AFS, infection and haematology) and other relevant stakeholder organisations such as laboratories performing IFI-related tests, Royal Colleges and specialist societies such as the British Infection Association, the British Society for Haematology, the British Society for Medical Mycology and the Royal Pharmaceutical Society.

Other core outputs from the trial will be:

- Quantitative and qualitative process evaluation data to inform the pathway to adoption and dissemination/implementation, and other AFS interventions and the wider AFS agenda
- A training, engagement and PPI legacy built around the development of a network of stakeholders interested in this aspect of AFS and the wider AFS agenda
- The results of this trial are likely to be practice changing/informing and are therefore highly likely to be incorporated into national and international guidelines
- Publications in high-impact open-access journals relating to the work packages as outlined
- Conference presentations at high-impact, relevant national and international conferences relating to the key components of the work packages: trial design, main trial, process evaluation and cost-effectiveness
- Cost-effectiveness data to inform the NHS about the value for money of the intervention
- A potential research resource for the global research community to perform further research relating to the stored blood samples with linked clinical data, as outlined above
- Development and use of a DOOR endpoint as an exploratory outcome to assess relevance within the context of this trial and AFS

A partnership has been agreed with the British Society for Antimicrobial Chemotherapy (BSAC) to help deliver key engagement and post-trial adoption, training, and implementation for example through the following:

- A BSAC hosted, bespoke networking/project website (E-forum) to facilitate and enhance sharing and communication of research outputs. Resources from webinars and training events will be housed on this site, providing key output legacy and reusable and updateable materials that are available beyond the projects timeframe
- Hosting of a national trial-related event and series of up to four separate webinars to promote dissemination of research outputs, stakeholder involvement and networking. Recordings of events will be hosted on the BSAC e-learning hub (https://www.infectionlearninghub.co.uk/)
- Development of an accredited e-learning course relating to project outputs. The course will be hosted on the FutureLearn Platform https://www.futurelearn.com/ and developed as a SCORM (Sharable Content Object Reference Model) compliant course to enable NHS trusts to download and deploy on local intranets
- Outputs of webinars and other meetings as potential leading articles in BSAC journals
- Appropriate use of social media to engage with the public, professionals and stakeholders

## Discussion

The use of empirical or preemptive systemic AFs in patients with AL and related conditions undergoing intensive chemotherapy is a controversial area of clinical practice with a sub-optimal high-quality evidence base to inform how we currently prescribe and order tests. In the UK NHS, heterogeneity in clinical practice in this area appears to be considerable with some centres performing systematic IFI biomarker monitoring while prescribing mould-acting antifungal prophylaxis while others do much less. This is unacceptable in the context of emerging antimicrobial resistance in fungi, as well as the associated fiscal costs, medication burden for patients, drug-drug interactions and adverse effects of AF agents. This trial aims to further the knowledge of strategies to safely optimise AF use and will generate the next step on the evidence ladder following the recent trial of Maertens et al. [12]; i.e. can antifungal prophylaxis be safely omitted by using a biomarker-based diagnostics approach in the prevention and treatment of IFI in a high-risk patient group.

The clinical and cost-effectiveness of two strategies will be compared: a biomarker-based AFS strategy, vs a prophylactic strategy (the current most commonly used SoC for these patients in the UK’s NHS). The trial has an inbuilt 9-month pilot phase and mixed methods process evaluation to assess the fidelity and feasibility of the trial and the intervention, and to inform post-trial implementation. The results will be disseminated through various relevant outputs in collaboration with stakeholders, including peer-reviewed publications and a legacy engagement website. Patient and public involvement was important in informing the design of the trial, including the choice of primary outcome.

The generated evidence will inform global clinical practice and approaches within the emerging discipline of antifungal stewardship, as well as improving knowledge about how to prescribe antifungals for optimal patient safety while minimising costs and emerging antifungal resistance.

## Trial status

The BioDriveAFS trial is working to protocol version 2.4 (12/02/2024). Recruitment began in September 2022 and is due to be complete by 30th August 2024.

### Supplementary Information

Additional file 1. BioDriveAFS Parallel Studies: Description of the planned additional research running in parallel to the main BioDriveAFS trial, with consenting main trial participants and certain participating sites. Consent to these are optional and will not impact on patients’ involvement in the main trial, or impact on their standard of care. 

Additional file 2. BioDriveAFS Mixed Methods Process Evaluation: A summary of further in-depth details regarding data collection, analysis methods, frameworks and outcomes. 

## Data Availability

The patient consent form will be inclusive of a statement of permission to access source data by staff working on the study, and for regulatory and audit purposes. This will be explicitly explained as part of the consent process and within the Participant Information Sheet. Upon completion of analysis and all intended outputs by YTU, anonymised data will be made available for meta-analysis, and for other authorised researchers and journals who request this data for publication purposes. The Chief Investigator and study sponsor will review all requests for access to data.

## Abbreviations

AE: Adverse events
AF: Antifungal
AFS: Antifungal Stewardship
aGVHD: Acute graft-versus-host disease
AL: Acute leukaemia
ALL: Acute lymphoblastic leukaemia
AML: Acute myeloid leukaemia
BAL: Broncho-alveolar lavage
BG: Beta-D-glucan (biomarker)
BSAC: British Society for Antimicrobial Chemotherapy
CFR: Case Report Form
CFIR: Consolidated Framework for Implementation Research
CNS: Central nervous system
CPG: Clinical Practice Guidelines
CRF: Case report form
CSF: Cerebrospinal fluid
DDD: Defined daily dose
DNA: Deoxyribonucleic acid
DMEC: Data Monitoring and Ethics Committee
DOOR: Desirability of Outcome Ranking
E-consent: Electronic informed consent
EORTC/MSG: European Organization for Research and Treatment of Cancer and the Mycoses Study Group
ESMO: European Society of Medical Oncology
EQ-5D-5L: EuroQol Quality of Life Measure
GDPR: General Data Protection Regulations
GM: Galactomannan (biomarker)
GCP: Good Clinical Practice
HRCT: High-resolution CT
HRMDS: High-risk myelodysplastic syndromes
HRQoL: Health-related quality of life
HIV: Human immunodeficiency virus
HRA: Health Research Authority
IA: Invasive aspergillosis
IC: Intensive chemotherapy
ICERs: Incremental cost-effectiveness ratios
IFI: Invasive fungal infection
IMD: Index of multiple deprivation score
MRI: Magnetic resonance imaging
NHS: National Health Service
PAG: Patient Advisory Group
PIL: Patient Information Leaflet
PI: Principal Investigator
PCR: Polymerase chain reaction
PCP: Pneumocystis pneumonia
QALY: Quality-adjusted life year
RCT: Randomised control trial
REC: Research Ethics Committee
RN: Research nurse
SAE: Serious adverse events
SAP: Statistical Analysis Plan
SCORM: Sharable Content Object Reference Model
SIV: Site Initiation Visit
SoC: Standard of Care
TAT: Turnaround time
TMG: Trial Management System
UoY: University of York
YTU: York Trials Unit
