# Supplementary material for: Biomarker Driven Antifungal Stewardship (BioDriveAFS) in acute leukaemia—a multi-centre randomised controlled trial to assess clinical and cost effectiveness: a study protocol for a randomised controlled trial
Source: Trials. 2024 Jun 28;25:427. doi: 10.1186/s13063-024-08272-w (PMC11214238; doi:10.1186/s13063-024-08272-w)
Supplement: Supplementary file 1 — Additional file 1. BioDriveAFS Parallel Studies: Description of the planned additional research running in parallel to the main BioDriveAFS trial, with consenting main trial participants and certain participating sites. Consent to these are optional and will not impact on patients’ involvement in the main trial, or impact on their standard of care. [file 13063_2024_8272_MOESM1_ESM.pdf]

Additional File 1. Parallel studies to the Biomarker Driven Antifungal Stewardship (BioDriveAFS) Trial

1. List of abbreviations

AF	Antifungal
AFS	Antifungal Stewardship
ALL	Acute Lymphoblastic Leukaemia
AML	Acute Myeloid Leukaemia
BDG	Beta-D-glucan (biomarker)
CI	Chief Investigator
CVC	Central Venous Catheter
GCP	Good Clinical Practice
GDPR	General Data Protection Regulations
GM	Galactomannan (biomarker)
HRMDS	High Risk Myelodysplastic Syndromes
HTA	Human Tissue Authority
HYMS	Hull York Medical School
IA	Invasive Aspergillosis
IC	Intensive Chemotherapy
ICF	Informed Consent Form
IFI	Invasive Fungal Infection
ISRCTN	International Standard Randomised Controlled Trials Number
IV	Intravenous
MAG	Metagenomic Assembled Genomes
NCBI	National Center for Biotechnology Information
NIHR	National Institute for Health Research
NF	Neutropenic Fever
NHS	National Health Service
NHS R&D	National Health Service Research & Development
PCA	Principle Component Analysis
PCoA	Principle Coordinate Analysis
PCR	Polymerase chain reaction
PI	Principal Investigator
PIC	Participant Identification Centre
PIS	Participant Information Sheet
RCT	Randomised Control Trial
SDV	Source Data Verification
SoC	Standard of Care
SOP	Standard Operating Procedure
SmPC	Summary of Product Characteristics
VOC	Volatile Organic Compounds
YTU	York Trials Unit

2. Studies Summary

Acute leukaemias are blood cancers treated with intensive chemotherapy (IC) and carry a high risk of invasive fungal infection (IFI). The National Institute for Health and Care Research (NIHR) funded BioDriveAFS trial looks to compare twice-weekly blood biomarker monitoring using galactomannan (GM) and beta-D-glucan (BDG) to primary antifungal (AF) prophylaxis (standard of care (SoC)). Specific sites will be invited to collect, with consent, additional patient samples such as skin and oral swabs, breath and blood, for the use in local and potentially multi-centre parallel studies. These parallel studies will run alongside the BioDriveAFS trial predominantly to provide data on the associations between patients' individual microbiome and clinical outcomes, including the development of IFI, and whether subsequent false positive and negative biomarker results can be predicted at baseline or during chemotherapy. These data will inform subsequent research and funding applications relating to IFI related personalised supportive care in acute leukaemia.

3. Background and Rationale

Acute leukaemias, such as AML, ALL and HRMDS are potentially curable with IC [1]. AML is the most common with over 3000 new cases diagnosed in the UK yearly [2]. IC results in 60% survival at two years, but 30-day treatment mortality is 4-6% due to the chemotherapy related prolonged pancytopenia and increased risk of neutropenic fever (NF) thereof [1]. Acute leukaemia patients receiving chemotherapy are at risk of IFI. IFI is mostly due to invasive aspergillosis (IA) of the lungs and is associated with high mortality [3].

Supportive care for patients with acute leukaemia is a range of interventions that optimise the patient experience and minimise symptoms of disease or the adverse effects of treatment. Infection related supportive care to date, by necessity, has been 'broad-brush', although it is known that some patients are at higher risk of infection complications than others [4]. It is increasingly recognised that the nature of a patient's microbiome, including the risk of infection, is associated with key disease outcomes [5-7], and influences the volatile organic compounds (VOCs) within human breath [8]. In an era of rapidly evolving clinical technologies, there is an opportunity to provide personalised supportive care, for example, those at highest risk of infection receiving more targeted support. The potential fundamental advantage of such an approach is that those at lower risk of infection could avoid unnecessary antimicrobial drug exposure with associated adverse effects [9] and drug-drug interactions [10], whilst optimising interventions for those at higher risk of life-threatening infections. Such an approach could also minimise the risk of microbiome disruption (by minimising drug exposure) and the further development of antimicrobial resistance [11], at both individual and environmental levels, which disproportionately impacts this group.

The BioDriveAFS trial will assess whether a serial biomarker-based diagnostic strategy (without AF prophylaxis), using the blood biomarkers of IFI, GM and BDG, is superior to prophylactic AFs and SoC (i.e. reactive tests) in reducing AF use in patients with acute leukaemia having IC, without adverse impact on health-related quality of life or IFI incidence. Diagnostic research in this area is rapidly evolving and it is also important to investigate other approaches in this patient group such as why some patients get a fungal infection and others do not and why the blood tests for fungal infections become positive in some patients but not others. Such research could lead to improved tests or treatments for patients.

These BioDriveAFS parallel studies described here involve collecting additional samples from a sub-set of patients in the main trial (control and intervention arms) who consent to this (voluntary without precluding involvement in the main trial) for storage and further research with the ability to link that research to the other data collected as part of the BioDriveAFS trial.

Aim: To deliver a programme of research, in parallel to the main BioDriveAFS trial, to establish the foundations for a personalised (individualised) approach to supportive care in acute leukaemia patients and the development of blood, microbiome, and breath-based predictive tools that can subsequently be experimentally applied and tested in larger, externally funded clinical studies.

Hypotheses:

1. The nature of a patient's microbiome and breath (VOCs) at baseline and during treatment are related and can predict key clinical outcomes, such as IFI, infection related mortality and remission status, in acute leukaemia patients undergoing IC, and thereby inform a personalised medicine approach to supportive care.
2. False negative / positive biomarker results for IFI (GM / BDG) can be predicted by clinical and/or biological patient characteristics that can be identified either at baseline or at the time of the blood test, or by further sample analysis.

4. Objectives

Objective 1

To investigate the clinical and biological patient and sample characteristics at baseline and/or at the time of the blood test, or by further sample analysis that predict false negative / positive blood biomarker results for IFI (GM / BDG). [BioDriveAFS trial centres that have been invited and are able and willing to participate in this parallel study]

Objective 2

To obtain and successfully transport, store, and analyse serial samples of both the human microbiome (skin, and oral) and VOCs of the human breath in a sub-cohort of acute leukaemia patients undergoing chemotherapy within the context of the BioDriveAFS trial [BioDriveAFS trial centres that have been invited and are able and willing to participate in this parallel study]

Objective 3

To investigate temporal associations between the human microbiome (skin and oral), exposure to antimicrobials/AF drugs, and clinical outcomes (e.g. episodes of IFI, response to chemotherapy, mortality) in a cohort of acute leukaemia patients undergoing IC within the context of the BioDriveAFS trial to provide pilot data for a future funding application for a larger study that will investigate the potential of microbiome surveillance to inform a personalised approach to medical intervention during IC (e.g. the requirement for AF drug prophylaxis at a particular timepoint). [BioDriveAFS trial centres that have been invited and are able and willing to participate in this parallel study]

Objective 4

To investigate the relationships between the human microbiome and VOCs within human breath to provide pilot data to inform a future funding application for a larger study to investigate the role of non-invasive, breath-based microbiome surveillance during chemotherapy. [BioDriveAFS trial centres that have been invited and are able and willing to participate in this parallel study]

Objective 5

To investigate the relationships between the serial nature of the different components of the human microbiome (e.g. skin and oral) in acute leukaemia patients undergoing IC (as above). [BioDriveAFS trial centres that have been invited and are able and willing to participate in this parallel study]

5. Consent and recruitment

Patients will be able to participate in the main BioDriveAFS trial without consenting to the parallel studies, with no impact on their involvement in the main BioDriveAFS trial or on the quality of their routine clinical care. If patients consent to additional samples being collected and used for the parallel studies, this will be documented on the main BioDriveAFS trial consent form. If the patient chooses to withdraw from this part of the study, they can request

that any stored samples are destroyed. Further details on the recruitment and consent process is given in the main BioDriveAFS protocol.

6. Study design and analysis

6.1. Microbiome analyses

Patients consenting to this parallel study will have non-invasive serial sparse (no more than twice monthly) sampling of, skin swabs, oral (swabs and wash) and breath samples before, during and after chemotherapy (at approximately 9 months aligned with clinic follow-up).

Skin/oral samples

Swabs will be taken from dry, moist, sebaceous and foot skin, the buccal mucosa, and an oral wash specimen. Samples will be collected from patients at baseline, as well as during and after each cycle of chemotherapy. Samples will be frozen at -80°C within 4 hours of collection and stored in approved secure Hospital or University premises.

DNA extraction, library preparation and sequencing

DNA will be extracted from each sample. Long fragment DNA sequencing will be performed using an Oxford Nanopore Technologies' PromethION where for each specimen equal quantities of DNA will be barcoded, pooled and sequenced. Constitutional (human) DNA will not be analysed so the issue of constitutional susceptibility to disease will not arise for either the participating individual or non-participating family members.

Sequence analysis

Using a custom bioinformatics pipeline the long reads will be quality filtered, and demultiplexed. Sequences will be classified by mapping them to a taxonomical database based on NCBI RefSeq. Analysis will be performed in statistical packages such as R. Relative abundance changes of microbes over treatment will be visualised and correlated with other clinical and biological marker measurements to determine the effect of treatments or other effects on patient microbiomes.

6.2. Breath (VOC) Analyses

Patient exhaled breath collection will be performed utilising commercially available products, which are designed specifically for breath research and are CE marked. Patients will breathe into these devices following the relevant product guidelines and VOC samples will be frozen and stored appropriately prior to analysis.

Sample Analysis

VOC data and clinical results will be correlated using a series of appropriate feature extraction processes and multivariate statistical analyses to visualise and explore the potential of VOCs as non-invasive clinical indicators for effective treatments or as diagnostics for off-target effects of treatments on patient microbiomes.

6.3. Blood samples

At participating sites, for patients in the intervention arm, an additional blood specimen will be taken at the same time as biomarker blood tests. This sample will be stored at -80°C to be used in future research studies following appropriate ethical approvals.

7. Data management

Data collected from sites will be entered, reconsolidated, stored, and archived as per the main BioDriveAFS protocol. Additional specimens for the purposes of these parallel studies will be identified by the participant's Unique Trial Number. Each site will hold data according to the General Data Protection Regulations (GDPR) and the Data Protection Act 2018, and study files will be stored securely in accordance with Good Clinical Practice (GCP) guidelines.

The additional samples will be held and processed in accordance with the Human Tissue Act 2004 and the Human Tissue (Scotland) Act 2006. Custody of the samples will be with the site principal investigator (PI) or collaborator (as appropriate) until the transfer of specimens (if this is required) to a central or collaborator laboratory in which case the collaborator or chief investigator will have custody (as appropriate). Samples will be disposed of in accordance with the Human Tissue Authority's Code of Practice at the end of research, no later than 5 years after the end of study completion.

8. Dissemination and projected outputs

Results from these studies will be presented at scientific conferences and submitted for thesis (some of this work will form the basis of the BioDriveAFS trial's Clinical Research Fellows' higher research degrees) and to peer-reviewed journals as appropriate. Results will also be reported via the main trial website in collaboration with the British Society for Antimicrobial Chemotherapy (BSAC).

9. References

1. Burnett AK, Russell NH, Hills RK, Kell J, Cavenagh J, Kjeldsen L, et al. A randomized comparison of daunorubicin 90 mg/m2 vs 60 mg/m2in AML induction: Results from the UK NCRI AML17 trial in 1206 patients. Blood. 2015 Jun 18;125(25):3878–85.
2. Cancer Research UK. Acute myeloid leukaemia (AML) incidence statistics | Cancer Research UK [Internet]. 2014.
3. Chen CY, Sheng WH, Tien FM, Lee PC, Huang SY, Tang JL, et al. Clinical characteristics and treatment outcomes of pulmonary invasive fungal infection among adult patients with hematological malignancy in a medical centre in Taiwan, 2008–2013. J Microbiol Immunol Infect. 2020 Feb 1;53(1):106–14.
4. Wong TY, Loo YS, Veettil SK, Wong PS, Divya G, Ching SM, et al. Efficacy and safety of posaconazole for the prevention of invasive fungal infections in immunocompromised patients: a systematic review with meta-analysis and trial sequential analysis. Sci Rep. 2020 Sep 3;10(1):1–11.
5. Song Y, Himmel B, Öhrmalm L, Gyarmati P. The Microbiota in Hematologic Malignancies. Curr Treat Options Oncol. 2020 Jan 1;21(1).
6. Shen X, Yao YF, Li JY, Li Y. Human mycobiome and diseases. Hua Xi Kou Qiang Yi Xue Za Zhi. 2019 Jun 1;37(3):314–9.
7. Bandara HMHN, Panduwawala CP, Samaranayake LP. Biodiversity of the human oral mycobiome in health and disease. Oral Dis. 2019 Mar 1;25(2):363–71.
8. White LP, Price JS. Recent advances and novel approaches in laboratory-based diagnostic mycology [Internet]. Vol. 7, Journal of Fungi. Multidisciplinary Digital Publishing Institute; 2021. p. 1–14.
9. Mohr J, Johnson M, Cooper T, Lewis JS, Ostrosky-Zeichner L. Current options in antifungal pharmacotherapy. Pharmacotherapy. 2008 May;28(5 PART 1):614–45.
10. Moriyama B, Henning SA, Leung J, Falade-Nwulia O, Jarosinski P, Penzak SR, et al. Adverse interactions between antifungal azoles and vincristine: Review and analysis of cases [Internet]. Vol. 55, Mycoses. Mycoses; 2012. p. 290–7.
11. Marr KA, Seidel K, White TC, Bowden RA. Candidemia in allogeneic blood and marrow transplant recipients: Evolution of risk factors after the adoption of prophylactic fluconazole. J Infect Dis. 2000 Jan 1;181(1):309–16.
