# Supplementary material for: Biomarker Driven Antifungal Stewardship (BioDriveAFS) in acute leukaemia—a multi-centre randomised controlled trial to assess clinical and cost effectiveness: a study protocol for a randomised controlled trial
Source: Trials. 2024 Jun 28;25:427. doi: 10.1186/s13063-024-08272-w (PMC11214238; doi:10.1186/s13063-024-08272-w)
Supplement: Supplementary file 2 — Additional file 2. BioDriveAFS Mixed Methods Process Evaluation: A summary of further in-depth details regarding data collection, analysis methods, frameworks and outcomes. [file 13063_2024_8272_MOESM2_ESM.pdf]

Additional File 2: BioDriveAFS Process Evaluation supplementary information.

Additional File 2, Table 1. *Descriptive data for the mixed methods Process Evaluation*

**Data type**	**Data collection method**	**Importance**
Data of key ward characteristics.	*A log by RNs for each of the 40 sites. Consisting of data on: hospital size/type, AML/HRMDS/ALL/tMPN patient throughput, geographical location, length of stay and any other important data.*	*Assist in the sampling of the eight sites for the qualitative work.*
Recording of tacit knowledge - 8 sites involved in qualitative work.	*Capture informal/ tacit knowledge to understanding context, implementation or fidelity. This is data that is useful to the process evaluation but which exists outside of the formal interviews, e.g., opportune phone calls, emails or face to face information collected during site visits. Knowledge collected via fieldnotes and a researcher reflections diary.*	*An example of how this data might be important is to understand the differing levels of site engagement in the set-up period of the trial.*
A documentary analysis of site AF policies of accepting and declining sites.	*Collected from sites via emailing clinicians who have been in contact with the YTU team asking whether they are willing to send policy documents. Not likely to be in public domain therefore we will ask for email consent statements for inclusion in analysis - request via email alongside a detailed explanation of the rationale for the analysis.*	*Enable an understanding of the diverse approach to AF treatment for patients, differences and similarities and whether AF policies for haematology patients at some sites are too prohibitive for clinical teams to take part in the trial.*

Additional File 2, Table 2. *Process Evaluation summary of data collection methods, sampling and time points*

**Component of process evaluation**	**Method**	**Participant**	**Sample size**	**Time point**
Data about ward characteristics	*Completion of baseline data log*	*RNs*	*One log per participating ward*	*During study set up at each site*
Fidelity assessment (quantitative)	*Data collected from patients' medical notes*	*Patients (no contact)*	*All intervention arm patients*	*Pilot and main trial*
Understanding context and exploring implementation (qualitative)	*Interviews or focus groups*	*Healthcare staff*	*80 overall*<br>*40 participants x2 (does not have to be same participants)*	*40 during pilot trial*<br>*40 during main trial*
Understanding context and exploring implementation (qualitative)	*Interviews*	*Patients*	*40 overall*<br>*20 participants x2 (does not have to be same participants)*	*20 during pilot trial*<br>*20 during main trial*
Understanding context (qualitative)	*Interviews and SIV recordings*	*Clinical leads of declining sites and all pre-SIV and SIV videos*	*8-10 sites overall (8-10 clinical leads)*<br>*SIV recordings of as many sites as relevant*	*Interviews during pilot trial*<br>*SIV recordings analysis during months 7-9 of pilot phase and months 10-12 of full trial*
Fidelity assessment (qualitative)	*Structured phone interviews*	*Lead clinician per site*	*10 sites overall*<br>*5 highest performing sites*<br>*5 lowest performing sites*	*Towards end of trial*
Recording of tacit knowledge	*Field notes*	*By proxy from healthcare staff and RNs*	*As many sites as relevant*	*Set up, pilot and main trial*
AF policies documentary analysis (descriptive qualitative data)	*Documentary analysis*	*AF policies from both accepting and declining sites*	*As many sites as relevant (both declining and accepting sites)*	*During months 7-9 of pilot phase and months 10-12 of full trial*

Additional File 2, Table 3. *Process Evaluation data analysis, frameworks, and outcomes – supplementary information*

Timepoint	Data type	Analysis and outcomes
Analysis phase 1 – pilot stage	Quantitative data (patient clinical records)	Basic descriptive statistical analysis paying attention to interim levels of adherence, difference between sites.
	Qualitative data (interview transcriptions and SIV videos)	A rapid descriptive, thematic analysis to generate headline themes, for example about commonalities and differences in site context, clinical systems, team set-up/relationships and reasons behind declining or accepting trial participation. Emerging factors regarding implementation and interim moderating factors.
	A mixed methods integration	To refine the treatment pathway/clinician training to improve adherence moving forward into the main trial.
Analysis phase 2 – towards the end of recruitment	Qualitative	1) A deductive analysis of data from both phases using all domains of the Consolidated Framework for Implementation Research (CFIR) to understand core implementation barriers and levers [1]. To develop a robust post-trial implementation strategy and package to encourage a 'soft landing' of the intervention in the wider NHS (if successful).<br><br>2) A second rapid, inductive descriptive analysis with two purposes a) a focus on whether the intervention is becoming normalised in the settings, or not, how/why b) generation of definitive moderating factors (e.g., intervention complexity) which may explain low or high fidelity at sites.
Analysis phase 3 – towards the end of the trial period	Quantitative	1) Analysis of the quantitative fidelity data for the main trial, using descriptive statistics (see trial protocol paper)<br><br>2) Fidelity assessment using a fidelity scoring matrix (see details on scoring matrix in trial protocol paper)
	Qualitative	1) Deductive qualitative analysis of phone interviews with the x5 highest and x5 lowest ranked sites regarding fidelity. Framework analysis of responses.<br><br>2) A dynamic logic model will be developed to explicate the theory of change of the intervention following its testing in the trial [2].

References

1. Mills, Thomas, Rebecca Lawton, and Laura Sheard. 2019. “Advancing Complexity Science in Healthcare Research: The Logic of Logic Models.” *BMC Medical Research Methodology* 19 (1): 1–11.

2. Tissot, Frederic, Samir Agrawal, Livio Pagano, Georgios Petrikkos, Andreas H. Groll, Anna Skiada, Cornelia Lass-Flörl, Thierry Calandra, Claudio Viscoli, and Raoul Herbrecht. 2017. “ECIL-6 Guidelines for the Treatment of Invasive Candidiasis, Aspergillosis and Mucormycosis in Leukemia and Hematopoietic Stem Cell Transplant Patients.” *Haematologica* 102 (3): 433.
